# Barriers and Facilitators to Risk Reduction of Cardiovascular Disease in Hypertensive Patients in Nigeria

**DOI:** 10.5334/aogh.4131

**Published:** 2023-12-13

**Authors:** Janet Adeola, Fiona Obiezu, Oluwakemi Odukoya, Ugonnaya Igwilo, Adewunmi Usinoma, Ehete Bahiru, Folasade P. May

**Affiliations:** 1 David Geffen School of Medicine at the University of California Los Angeles, Los Angeles, California, USA; 2 Department of Community Health and Primary Care, College of Medicine, University of Lagos and Lagos University Teaching Hospital, Nigeria; 3 Department of Cardiology, David Geffen School of Medicine at the University of California Los Angeles, Los Angeles, California, USA; 4 Vatche and Tamar Manoukian Division of Digestive Diseases, Department of Medicine, David Geffen School of Medicine, University of California Los Angeles, Los Angeles, California, USA; 5 UCLA Center for Cancer Prevention and Control Research, UCLA Kaiser Permanente Center for Health Equity and Department of Health Policy and Management, Jonsson Comprehensive Cancer Center, Los Angeles, California, USA

**Keywords:** Hypertension, Cardiovascular Disease, Sub-Saharan Africa, Nigeria

## Abstract

**Background:**

In Sub-Saharan Africa (SSA), the prevalence of hypertension is increasing due to many factors like rapid population growth, globalization, stress, and urbanization. We aimed to characterize the perceptions of cardiovascular disease (CVD) risk among individuals with hypertension living in Nigeria and identify barriers and facilitators to optimal hypertension management.

**Methods:**

This cross-sectional survey study was conducted at a large teaching hospital in Lagos, Nigeria. We used a convenient sample of males and females, aged 18 or older, with a diagnosis of hypertension who presented for outpatient visits in the cardiology, nephrology, or family medicine clinics between November 1 and 30, 2020. A semiquantitative approach was utilized with a survey consisting of closed and open-ended questionnaires focused on patient knowledge, perceptions of CVD risk, and barriers and facilitators of behavioral modifications to reduce CVD risk.

**Results:**

There were 256 subjects, and 62% were female. The mean age was 58.3 years (standard deviation (SD) = 12.6). The mean duration of the hypertension diagnosis was 10.1 years. Most participants were quite knowledgeable about hypertension; however, we observed some knowledge gaps, including a belief that too much “worrying or overthinking” was a major cause of hypertension and that an absence of symptoms indicated that hypertension was under control. Barriers to hypertension management include age, discomfort or pain, and lack of time as barriers to exercise. Tasteless meals and having to cook for multiple household members were barriers to decreasing salt intake. Cost and difficulty obtaining medications were barriers to medication adherence. Primary facilitators were family support or encouragement and incorporating lifestyle modifications into daily routines.

**Conclusion:**

We identified knowledge gaps about hypertension and CVD among our study population. These gaps enable opportunities to develop targeted interventions by healthcare providers, healthcare systems, and local governments. Our findings also help in the promotion of community-based interventions that address barriers to hypertension control and promote community and family involvement in hypertension management in these settings.

## Introduction

Countries in Sub-Saharan Africa (SSA) face the concurrent burden of a high prevalence of communicable diseases and increasing rates of chronic non-communicable diseases (NCDs) [1]. This challenge is greatest in Low- to Middle-Income Countries (LMICs), where 78% of deaths from NCDs occur [2]. Cardiovascular diseases (CVD) account for a high proportion of these deaths, at 44% of NCD deaths annually [2]. Hypertension is one of the major risk factors for CVD [3]. It is associated with coronary heart disease, chronic kidney disease, and ischemic and hemorrhagic strokes, among other conditions [2]. Globally, hypertension accounts for at least 51% of deaths due to stroke and 45% of deaths due to heart disease [4].

According to the World Health Organization (WHO), the prevalence of hypertension is highest in Africa at 27% and has been increasing over the past few decades [2]. Previously published studies have examined hypertension prevalence, awareness of hypertension, and factors associated with blood pressure control [5,6,7]. A meta-regression modeling study using United Nations population demographics to determine the prevalence and incidence of hypertension in Africa estimated over 54.6 million cases in 1990 (19.1%), 92.3 million cases in 2000 (24.3%), 130.2 million cases in 2010 (25.9%), and a projected increase to 216.8 million cases by 2030 (25.3%) [8]. In SSA, the prevalence of hypertension is increasing due to many factors, such as rapid population growth, globalization, and urbanization [9]. Globalization has contributed to this trend by increasing the prevalence of risk factors for hypertension, including sedentary lifestyles and alcohol use [9]. Urbanization and economic development have also contributed to the emergence of a nutritional transition characterized by high-calorie, high-fat diets [10].

Studies examining the predictors of hypertension in Sudan and Eritrea found that lack of awareness, economic barriers, stress, lack of medication adherence, use of traditional remedies, and misconceptions about lifestyle modifications were barriers to hypertension management, while knowledge about the disease, family support, and governmental support were important facilitators to optimal management [6,7]. A South African study looking at the management of hypertension in an urban setting showed a need to improve knowledge and nutrition in these communities [11]. Data are otherwise limited, especially in Western Africa, where globalization and urbanization are rampant. In this study, we aimed to characterize perceptions of cardiovascular disease (CVD) risk, susceptibility, and severity among individuals living with hypertension in Nigeria. We also aimed to identify barriers and facilitators to optimal hypertension management.

## Methods

### Study design

We performed a clinic-based, cross-sectional study at the Lagos University Teaching Hospital (LUTH) in Lagos, Nigeria. The study was approved by UCLA’s institutional review board (IRB #20-0 00443) and LUTH’s Health Research Ethics Committee (ADM/DCST/HREC/APP/3877). Prior to participating in the study, participants were provided with a study information sheet, questions about the study were answered, and informed consent was obtained from each participant before completing the questionnaire. Ethical standards were maintained as outlined in the Helsinki Declaration.

### Study population

The study population was a convenient sample of males and females 18 or older diagnosed with hypertension who were chosen at random. All participants presented to an outpatient visit in the cardiology, nephrology, or family medicine clinics at least once between November 1–30, 2020. A sample size was calculated at the time the study was designed using a corrected Cochran’s (1977) formula [12]. A sample size of 91 was calculated based on a 95% confidence interval, Z = 1.96; expected prevalence/proportion of hypertension estimated at 38.1%(0.381) based on a previous study by Odili et al. (2020) [13]; marginal error was 10%; and population estimate was 24.6 million in Lagos, Nigeria [14]. All 256 participants were included in the final analysis, resulting in a marginal error of 5.95%(0.0595). Given the large number of qualitative/open-ended questions in this study, data was also collected until saturation was reached.

### Survey development and administration

The survey instrument included 54 Likert scale, free response, yes/no, and multiple-choice format questions intended for in-person one-on-one administration (Supplement). Questions focused on patient knowledge and perceptions of hypertension and CVD risk, as well as barriers and facilitators of behavioral modifications to reduce CVD risk. We also collected demographic data from participants, including date of birth, sex, educational attainment, health insurance status, and cost of personal hypertensive medications.

Several medical students were trained as data collectors to conduct the surveys in person, with interviews typically lasting between 20 and 30 minutes. Responses were documented in standardized data collection forms within Excel. Participants were compensated with a N500 (the equivalent of 1.22 United States Dollars (USD)) recharge card (a common prepaid card used to pay for telephone calls; it covers approximately one 50-minute local telephone call).

A literature review informed survey items from similar studies [15,16,17]. Some relevant survey questions were adapted from validated survey instruments, including the World Health Organization Quality of Life assessment (WHOQOL-100) [18] and the National Health Service (NHS) Health Check Programme Questionnaire [19]. Study materials, including the survey script, eligibility screening form, oral consent form, and study information sheet, were pilot tested with non-study patients prior to the study initiation. We also pilot-tested the study survey between October 2019 and November 2020. All piloted materials were modified to ensure cultural appropriateness, clarity, and comprehension.

### Data Analysis

We used SPSS version 25 to determine means, standard deviations, frequencies, percentages, and ranges for survey responses. We used thematic analysis for the 11 free response survey items, which involved reading through all responses, coding responses for overarching themes and subthemes, and extracting representative text (Table S1). After review, the study team finalized a list of themes, subthemes, and identified quotes.

## Results

### Survey participation

Of the 260 participants who filled out the survey, 256 (98.5%) were included in the final analysis. Four of the participants were excluded due to an unclear diagnosis of hypertension and completing less than 80% of the survey.

### Demographics of the study sample

The majority of participants were female (n = 157, 62.0%), and the average age of all participants was 58.3 years (SD = 12.6; range 25–92 years). Over half (n = 153, 59.8%) of the participants completed at least university, college, or polytechnic studies at the highest level of education.

The mean duration of hypertension among this population was 10.1 years, and 58.2% (n = 149) reported visiting their physician more than three times a year. Most participants did not have health insurance (n = 203, 79.3%), and 229 (89.5%) reported spending an average of N12,099 (the equivalent of 29.21 USD) per month on antihypertensive medications (Table 1).

**Table 1:** Demographic and clinical characteristics of the study sample, N = 256.


PARTICIPANT CHARACTERISTICS	N(%), MEAN (SD), OR RANGE

Age N (%)	250 (97.7%)

Mean (SD)	58.3 (12.6)

Range	25–92

Sex	

Male	97 (37.9%)

Female	157 (61.3%)

Unknown	2 (0.8%)

Insurance status	

Insured	49 (19.1%)

Uninsured	203 (79.3%)

Unknown	4 (1.6%)

Highest level of formal education	

No formal education	8 (3.1%)

Completed primary school	33 (12.9%)

Completed junior secondary school	10 (3.9%)

Completed senior secondary school	49 (19.1%)

Completed university, college, or polytechnic	153 (59.8)

Unknown	3 (1.2%)

Duration of hypertension diagnosis (years) N = 254	

Mean (SD)	10.11 (9.232)

Range	0.5–54

Hypertension medication cost (Naira) N = 229	

Mean (SD)	12,099 (13,599)

Range	0–100,000

Frequency of physician visits	

Never	13 (5.1%)

Once a year	22 (8.6%)

2–3 times a year	71 (27.7%)

More than 3 times a year	149 (58.2%)

Unknown	1 (0.4%)

Tobacco Use	

Yes	4 (1.6%)

No	252 (98.4%)

Alcohol Use	

Yes	30 (11.7%)

No	225 (87.9%)

Unknown	1 (0.4%)

Advised to lose weight	

Yes	124 (48.4%)

No	129 (50.4%)

Unknown	3 (1.2%)

Exercise at least 20 min daily	

Yes	200 (78.1%)

No	53 (20.7%)

Unknown	3 (1.2%)


### Hypertension knowledge and control

Participants were asked “What does hypertension mean to you?” as the survey’s first question. The majority (n = 140, 54.7%) identified that hypertension means an elevation in blood pressure and/or an impairment in blood flow related to unhealthy dietary and sedentary lifestyles. They also attributed several symptoms to their hypertension, including fatigue, difficulty breathing and sleeping, dizziness, headaches, and leg swelling. However, when asked if the absence of symptoms indicates that hypertension is under control, 129 (50.4%) participants agreed or strongly agreed, whereas 87 (34.0%) participants disagreed or strongly disagreed with this statement. Nine (3.5%) participants characterized hypertension as a sudden and silent killer.

**Figure 1:**
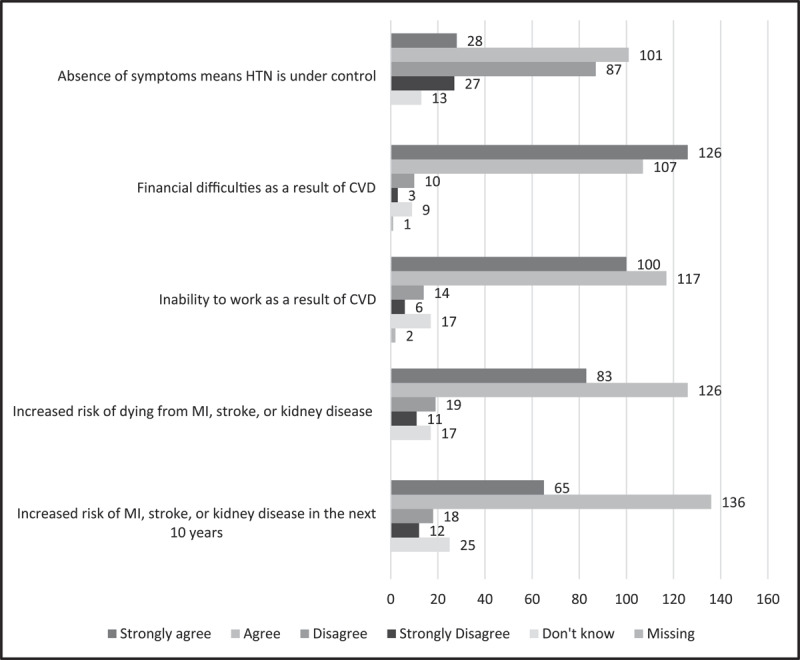
Perceptions of hypertension related risk for cardiovascular disease among survey participants, N = 256.

Several items on the survey also measure participants’ perceived susceptibility and severity of CVD risk. Most participants believed that tobacco use (n = 218, 85.2%), alcohol use (n = 239, 93.4%), being heavy or overweight (n = 239, 93.4%), lack of exercise (n = 236, 92.2%), and high salt intake (n = 249, 97.3%) make them more susceptible to hypertension. Furthermore, 217 (84.8%) participants agreed or strongly agreed that an individual who suffered a heart attack, stroke, or kidney disease as a consequence of hypertension would not be able to work as they usually do, while 233 (91.0%) agreed or strongly agreed that they would experience financial difficulty. Most participants also agreed or strongly agreed that hypertension increases one’s risk of developing a heart attack, stroke, or kidney disease in the next 10 years (n = 201, 78.5%) and dying from these complications (n = 209, 81.6%) (Figure 1).

When asked about strategies for achieving hypertension control, participants reported the importance of medication adherence and lifestyle modifications, as illustrated by one participant who stated, “*I use my drugs diligently and faithfully*” (Table S1). As for lifestyle modifications, 56 (21.9%) participants believed stress and anxiety; specifically, “*worrying and overthinking*” made it difficult to control their hypertension. For example, one participant stated that “*lack of relaxing my mind or overthinking*” contributed to difficulty managing his hypertension (Table S1). In addition, 76 (29.7%) participants reported that they made it a daily practice to reduce stress to help control their hypertension, with participants reporting limiting behaviors that involved stress and anxiety such as heavy labor, stressful jobs, worrying, excessive exercise, agitation, and anger.

### Lifestyle and behavioral modifications related to hypertension control

Most participants (n = 254, 99.2%) agreed that lifestyle modifications could help lower blood pressure. In addition to medication adherence, participants identified physician appointment compliance, home blood pressure monitoring, stress reduction, sleep hygiene, exercise, and diet modification (including a low-salt diet) as effective ways to manage their hypertension. Some participants (n = 10, 3.9%) also endorsed herbal medication use.

**Figure 2:**
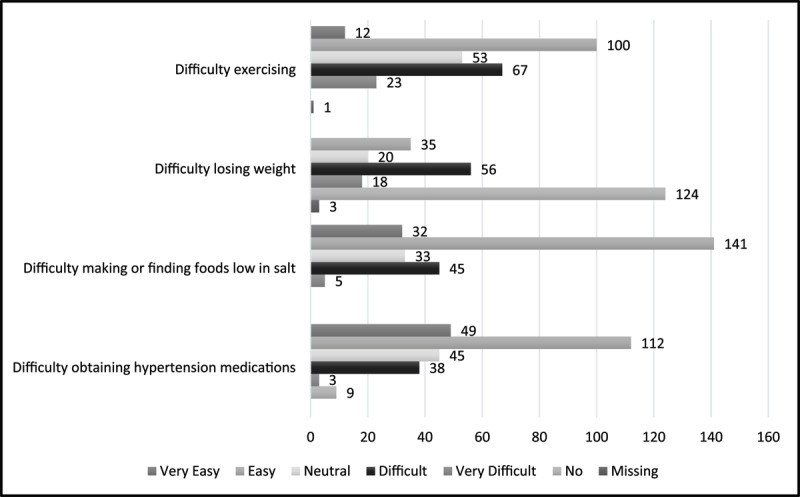
Perceptions of exercise, weight loss, low salt diet and access to hypertensive medications among survey participants, N = 256.

Ninety-eight percent (n = 252) of participants reported that they do not smoke tobacco or consume alcohol (n = 226, 88.2%). Forty-three percent (n = 112) of participants reported they found it easy or very easy to exercise, 35.2% (n = 90) found exercise difficult or very difficult, and 20.7% (n = 53) were neutral. Forty-eight percent (n = 124) had not been advised by a health professional to lose weight or had attempted to lose weight. Of those who had been advised to and attempted to lose weight, 13.7% (n = 35) found it easy to lose weight, compared to 28.9% (n = 74) who reported that weight loss was difficult or very difficult. Sixty-seven percent (n = 173) of participants found it easy or very easy to make or find foods low in salt; 12.9% (n = 33) were neutral; and 19.5% (n = 50) found it difficult or very difficult. Sixty-two percent (n = 161) of people found it easy or very easy to obtain hypertensive medications, whereas 16.0% (n = 41) people found it difficult or very difficult, and 17.6% (n = 45) were neutral (Figure 2).

### Barriers to hypertension management

**Figure 3:**
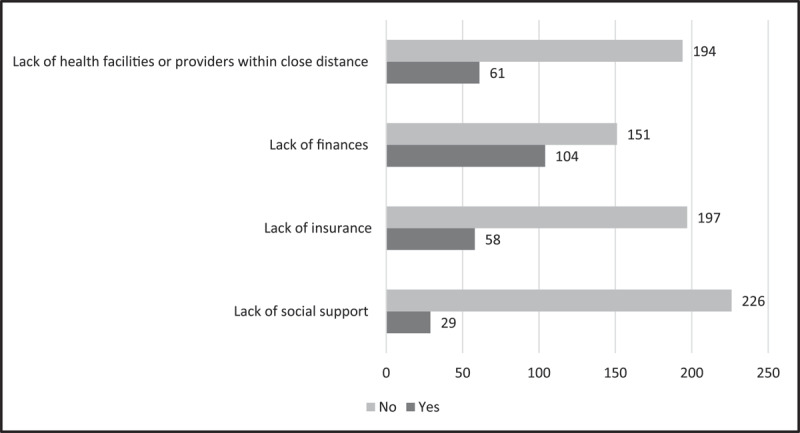
Reported factors that prevent self-management of hypertension among survey participants, N = 256.

The reported barriers to hypertension management are described in Table S1, with supporting examples from study participants. Of note, participants did not believe that lack of health facilities within close proximity (n = 194, 75.8%), lack of finances (n = 151, 59.0%), lack of insurance (n = 197, 77.0%), or lack of social support (n = 226, 88.3%) prevented them from controlling their hypertension (Figure 3).

#### Age, discomfort/pain, and lack of time as barriers to exercise and weight loss

Participants stated that aging, pain, and discomfort, as well as concerns about the worsening of pre-existing arthritis and other ailments, made it hard to walk long distances or consistently exercise: “*serious body pain, due to the stress of trekking [to journey long distances on foot]*” (Table S1). Another participant stated, “*I can’t stand for 2–3 hours in a day as recommended by the doctor. The exercise is too strenuous for me…. The walking is not easy*.” Lack of time was another barrier for participants to adhere to lifestyle modifications. Participants stated that it was difficult to find the time to exercise after a long day at work and to incorporate lifestyle changes into their daily routine.

#### Cost and difficulty obtaining medications as a barrier to medication adherence

Participants expressed that the “*unavailability of finance to buy the drugs and take it*” was a barrier to medication adherence (Table S1). Overall, participants described times when they simply did “*not have enough money to buy drugs*” (Table S1). Participants who did have health insurance (n = 49, 19.1%), expressed concerns about the unavailability of medications at pharmacies where their insurance was accepted. Participants also expressed a fear of receiving fake or expired medications.

When medications were obtained, barriers to regular use of the medications included not taking the medications if they felt well, feeling overwhelmed with too many medications or multiple doses, and forgetfulness.

### Facilitators to hypertension management

**Figure 4:**
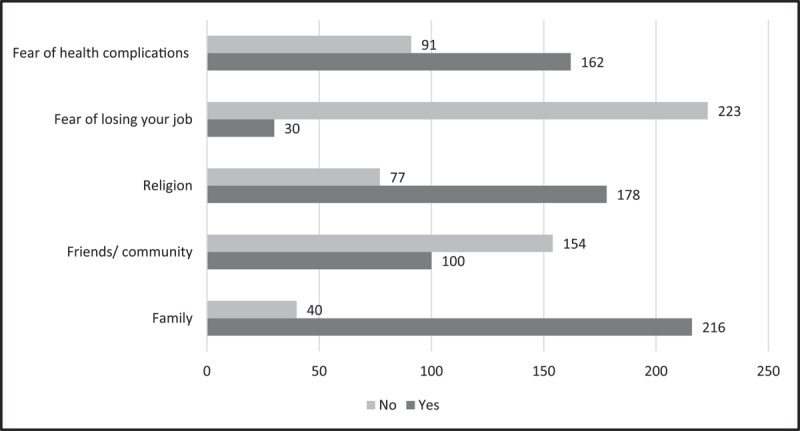
Reported factors that motivate self-management of hypertension among survey participants, N = 256.

Overall themes for facilitators of hypertension management are summarized in Table S1 with supporting participant statements. Sixty-three percent (n = 162) of participants believed that the fear of health complications and their financial implications were facilitators for achieving blood pressure control (Figure 4). Eighty-four percent (n = 216) of participants believed that their family motivated them to control their hypertension, while 69.5% (n = 178) believed their religion was a motivator (Figure 4).

#### Family support and encouragement as facilitators for adherence to medications and lifestyle modification

Of the participants, 84% (n = 216) cited family as a motivator for achieving blood pressure control. Participants expressed that they received help from family members to run errands, purchase medications, prepare low-salt meals, and encourage them to adhere to medications and lifestyle modifications. Regarding preparing low-salt meals, one participant noted that *“once the family is aware of the health status, they help to prepare it adequately”* (Table S1). Others expressed that their family members showed support by also eating low-salt meals. Of the 50 (19.5%) participants who found it difficult to make or eat low-salt meals, those responsible for cooking for their family found it difficult to make food with less salt, given that their family would not enjoy the taste of these meals. Others expressed that their meals were either bought from restaurants or prepared by other family members who may not consider their health concerns when seasoning meals (Table S1).

#### Creating a routine and incorporating lifestyle modifications into daily activities as a facilitator to hypertension management

Some participants expressed that creating a daily routine facilitated adherence to medications (n = 50, 19.5%), exercise (n = 89, 34.8%), and a low-salt diet (n = 38,14.8%). Participants noted that daily participation in walking to and from work, completing chores around the home, and physical labor at work made it easier to exercise regularly. One participant mentioned that they “*normally do a lot of trekking*” (Table S1) as part of their daily schedule, and another mentioned that “*it [daily physical activity] helps to keep me in shape and also maintain a healthy posture and a good weight*” (Table S1). Overall, participants acknowledged that adherence to lifestyle modifications, stress reduction strategies, and medication adherence were all necessary for good health and well-being.

## Discussion

In this study, we examined perceptions of CVD risk and barriers and facilitators to hypertension management among individuals living with hypertension in Nigeria. We found that most participants were quite knowledgeable about hypertension and believed it was a serious condition that could make the individual susceptible to CVD. However, we identified some gaps in knowledge, including a belief that too much “worrying or overthinking” was a major cause of hypertension and that an absence of symptoms indicated hypertension was under control. Additionally, our data revealed that the main barriers to hypertension management were age, discomfort or pain, and lack of time as barriers to exercise and weight loss; tasteless meals and cooking for multiple household members as barriers to decreasing salt intake; and cost or difficulty obtaining medications as barriers to medication adherence. The main facilitators of hypertension management included family support and encouragement and incorporating lifestyle modifications like exercise into daily routines.

Limited knowledge about hypertension has been previously identified as a barrier to management in SSA [11,20,21,22,23]. In our study, however, most participants were knowledgeable about hypertension, its risk factors, symptoms, necessary lifestyle modifications, and CVD complications. In fact, most participants identified fear of complications as a major motivator for hypertension management. This finding may be related to the high level of education among our study participants or the study’s urban setting. Similar findings were noted in another study where participants in Delta State, Nigeria, were found to have good knowledge of and attitudes towards hypertension [24]. Participants were also clinic patients who likely received more hypertension-related information than the typical layperson.

Regarding contributors to hypertension, there was a recurrent theme that too much “worrying or overthinking” was a major cause of hypertension, a finding that is consistent with a study in Tanzania in which all participants believed that “thinking too much” was the major, if not the sole, cause of their hypertension and that one could control hypertension by “reducing thinking” [25]. Although stress and anxiety may lead to relative increases in blood pressure, and their reduction can be additive in hypertension management, stress and anxiety are not the primary or sole causes of uncontrolled hypertension, nor is their reduction the primary treatment [25,26], as was believed by participants in our sample, indicating a knowledge gap. Additionally, half of our participants assumed that the absence of symptoms indicated that their hypertension was under control. This sentiment translates to difficulty with medication adherence, with some participants stating that they do not take their medications if they feel well. Other studies have documented a lack of knowledge that hypertension is often symptomless [20,27,28,29].

Additional important findings included knowledge gaps about symptoms and physical activity. Also, similar to our findings, prior studies have demonstrated misconceptions about physical activity, with some participants believing that they were too old for physical activity or that it would worsen their current ailments, while others complained of a lack of time and competing priorities [7,30,31].

The most commonly reported barriers to hypertension management were concerns about tasteless meals and having to cook for multiple family members as an obstacle to reducing salt intake and financial barriers to purchasing medications, equipment (e.g., home blood pressure cuffs), and recommended foods [7,32,33,34,35,36,37]. A review of similar studies revealed that Nigerians with hypertension spend up to 10% of their income on treatment and that improved consumer awareness about healthy salt substitutes that are cheaply available can help patients [38,39]. The use of traditional or alternative medicine and spiritual therapies was not a big theme of our study compared to others [7,20,40]. However, many of our participants did indicate that religion was a motivator for hypertension management. This variation may be due to the urban setting, high education among our participants, or the desire to provide only medically acceptable answers in the clinic where they received health care.

A study conducted in Eritrea found that patient-level barriers to hypertension management include stress from a fear of complications and not wanting to burden one’s family [8]. In our study, this factor was viewed as a facilitator, with participants being motivated to adhere to medications and lifestyle modifications so as not to burden their families with complications from their hypertension. However, as in the Eritrean study and others, family involvement was mentioned as a community-level facilitator [7,32,33,38,41,42]. Participants said that their family members provided assistance with food choices and running errands (e.g., transportation to appointments, purchasing medications), making meals, appointments, and medication reminders, and participating in lifestyle modifications (e.g., low-salt diet) [7,32,33,38,41,42]. Notably, additional facilitators include creating a daily routine for healthy lifestyle modifications. Examples include integrating exercise into daily activities and chores such as walking to and from work, farming, and engaging in household chores.

Our study had some limitations, one of which was that it was conducted in one large public hospital in Lagos, Nigeria. Therefore, this study may not be generalizable to the whole of Nigeria or SSA, individuals living in rural settings, or individuals who do not frequent large hospitals. Also, given the study’s setting, participants may have provided socially desirable responses about their health behaviors. Finally, we did not assess the perspective of family members or healthcare providers in this study.

Despite these limitations, the study has several strengths. First, using a survey design, we characterized the perceptions of CVD risk, susceptibility, and severity among individuals with hypertension living in Nigeria. We also provided a detailed description of barriers and facilitators to optimal hypertension management in this setting, which has not been done previously. Second, the interview script was based on a literature review, feedback from native experts, and the results of our pilot testing, further expanding upon previous research on this topic. Another strength is that interviews were conducted by trained data collectors who were also Nigerian, thus able to engage participants in detailed face-to-face discussions, identify nuances in responses, and probe unclear answers in real-time. Our study represents a true global health research partnership between an academic institution in the United States and a comprehensive clinic in Nigeria. Local team members in Nigeria played a critical role in the study’s design, implementation, and interpretation.

## Conclusion

Overall, the results of this study suggest that despite a high level of knowledge among participants, there are some knowledge gaps about hypertension and CVD among patients who are affected by these conditions in Nigeria. These gaps can be addressed with interventions by health care providers, health care systems, and local governments to provide more targeted education about these highly prevalent and increasingly impactful conditions in LMICs. Collaborations between healthcare providers and local communities could yield specific strategies for conveying essential information on hypertension and CVD management. On a governmental level, strategies to reduce hypertension include product reformulation towards low-sodium foods, improving consumer awareness and education [43], and cost-reduction strategies for healthy food items and medications. Findings from this study also underscore the importance of family support and encouragement in hypertension management. Further research should focus on exploring effective ways to strengthen family involvement in patients’ CVD risk management by utilizing important cultural events or religious gatherings for education and the development of support networks. Implementing community-based interventions that address barriers to hypertension control with a focus on promoting community and family involvement may be particularly impactful in these settings.

## Data Accessibility Statement

The datasets used and analyzed during the current study are available from the corresponding author upon reasonable request.

## Additional File

The additional file for this article can be found as follows:

10.5334/aogh.4131.s1 Supplement. Table S1 Summary of survey domains, survey themes, and examples of participant responses.

## Figures and Tables

**Table:** 

SSA	Sub-Saharan Africa
CVD	Cardiovascular Disease
LMIC	Low to Middle-Income Countries
WHO	World Health Organization
LUTH	Lagos University Teaching Hospital
WHOQOL-100	World Health Organization Quality of Life assessment (100 questions)
NHS	National Health Service
NCDs	Non-Communicable Diseases
SD	Standard Deviation
USD	United States Dollar

## References

[1] Kushitor MK, Boatemaa S. The double burden of disease and the challenge of health access: Evidence from Access, Bottlenecks, Cost and Equity facility survey in Ghana. PLoS ONE. 2018; 13(3): e0194677. DOI: 10.1371/journal.pone.0194677 29570720 PMC5865721

[2] World Health Organization. Noncommunicable diseases country profiles. World Health Organization. Published 2018. Accessed August 16, 2022. https://apps.who.int/iris/rest/bitstreams/1151362/retrieve.

[3] Wu CY, Hu HY, Chou YJ, Huang N, Chou YC, Li CP. High blood pressure and all-cause and cardiovascular disease mortalities in community-dwelling older adults. Medicine (Baltimore). 2015; 94(47): e2160. DOI: 10.1097/MD.0000000000002160 26632749 PMC5059018

[4] World Health Organization. A global brief on hypertension: silent killer, global public health crisis. World Health Day 2013. World Health Organization. Published 2013. Accessed August 16, 2022. https://apps.who.int/iris/handle/10665/79059.

[5] Bosu WK. The prevalence, awareness, and control of hypertension among workers in West Africa: A systematic review. Glob Health Action. 2015; 8: 26227. DOI: 10.3402/gha.v8.26227 25623611 PMC4306751

[6] Babiker FA, Elkhalifa LA, Moukhyer ME. Awareness of hypertension and factors associated with uncontrolled hypertension in Sudanese adults. Cardiovasc J Afr. 2013; 24(6): 208–212. DOI: 10.5830/CVJA-2013-035 24217260 PMC3767941

[7] Gebrezgi MT, Trepka MJ, Kidane EA. Barriers to and facilitators of hypertension management in Asmara, Eritrea: Patients’ perspectives. J Health Popul Nutr. 2017; 36(1): 11. DOI: 10.1186/s41043-017-0090-4 28407794 PMC5390439

[8] Adeloye D, Basquill C. Estimating the prevalence and awareness rates of hypertension in Africa: A systematic analysis. PLOS ONE. 2014; 9(8): e104300. DOI: 10.1371/journal.pone.0104300 25090232 PMC4121276

[9] Oyewole OE, Atinmo T. Nutrition transition and chronic diseases in Nigeria. Proc Nutr Soc. 2015; 74(4): 460–465. DOI: 10.1017/S0029665115002402 26242780

[10] BeLue R, Okoror TA, Iwelunmor J, et al. An overview of cardiovascular risk factor burden in sub-Saharan African countries: A socio-cultural perspective. Glob Health. 2009; 5: 10. DOI: 10.1186/1744-8603-5-10 PMC2759909 19772644

[11] Nkosi NG, Wright SC. Knowledge related to nutrition and hypertension management practices of adults in Ga-Rankuwa day clinics. Curationis. 2010; 33(2): 33–40. DOI: 10.4102/curationis.v33i2.1083 21469514

[12] Bartlett JE, Kotrlik JW, Higgins CC. Organizational research: Determining appropriate sample size in survey research. Information Technology, Learning, and Performance Journal. 2001; 19(1): 43–50. DOI: 10.5032/jae.2002.03001

[13] Odili AN, Chori BS, Danladi B, Nwakile PC, et al. Prevalence, awareness, treatment and control of hypertension in Nigeria: Data from a nationwide survey 2017. Glob Heart. 2020; 15(1): 47. DOI: 10.5334/gh.848 32923341 PMC7427662

[14] Lagos State Ministry of Science and Technology. About Lagos. Lagos State Government. Published 2023. https://lagosstate.gov.ng/about-lagos/ (Accessed: 21 September 2020).

[15] Oliveria SA, Chen RS, McCarthy BD, Davis CC, Hill MN. Hypertension knowledge, awareness, and attitudes in a hypertensive population. J Gen Intern Med. 2005; 20(3): 219–225. DOI: 10.1111/j.1525-1497.2005.30353.x 15836524 PMC1490067

[16] Perera M, de Silva CK, Tavajoh S, et al. Patient perspectives on hypertension management in health system of Sri Lanka: A qualitative study. BMJ Open. 2019; 9(10): e031773. DOI: 10.1136/bmjopen-2019-031773 PMC6797394 31594895

[17] Shrestha S, Shrestha A, Koju RP, et al. Barriers and facilitators to treatment among patients with newly diagnosed hypertension in Nepal. Heart Asia. 2018; 10(2): e011047. DOI: 10.1136/heartasia-2018-011047 30233660 PMC6135456

[18] World Health Organization. Programme on mental health: WHOQOL user manual, 2012 revision. World Health Organization. Published 2012. Accessed August 16, 2022. https://apps.who.int/iris/handle/10665/77932.

[19] Woringer M, Nielsen JJ, Zibarras L, et al. Development of a questionnaire to evaluate patients’ awareness of cardiovascular disease risk in England’s National Health Service Health Check preventive cardiovascular programme. BMJ Open. 2017; 7(9): e014413. DOI: 10.1136/bmjopen-2016-014413 PMC5623403 28947435

[20] Brathwaite R, Hutchinson E, McKee M, Palafox B, Balabanova D. The long and winding road: A systematic literature review conceptualising pathways for hypertension care and control in low- and middle-income countries. Int J Health Policy Manag. 2022; 11(3): 257–268. DOI: 10.34172/ijhpm.2020.105 32702800 PMC9278472

[21] Boateng D, Wekesah F, Browne JL, et al. Knowledge and awareness of and perception towards cardiovascular disease risk in sub-Saharan Africa: A systematic review. PloS One. 2017; 12(12): e0189264. DOI: 10.1371/journal.pone.0189264 29232703 PMC5726714

[22] Chimberengwa PT, Naidoo M; cooperative inquiry group. Knowledge, attitudes and practices related to hypertension among residents of a disadvantaged rural community in southern Zimbabwe. PLoS One. 2019; 14(6): e0215500. Published 2019 Jun 25. DOI: 10.1371/journal.pone.0215500 31237883 PMC6657811

[23] Kardoudi A, Kaoutar K, Chetoui A, Boutahar K, et al. Knowledge, attitudes and practices determinant’s regarding hypertension in Moroccan hypertensive patients. The Annals of Clinical and Analytical Medicine. Published online 2021. DOI: 10.4328/ACAM.20660

[24] Aghoja OC, Okinedo PO, Odili VU. Knowledge, attitude and practice of hypertensive patients towards hypertension in a secondary health care facility in Delta State. UK Journal of Pharmaceutical Biosciences. 2017; 5(2): 24. DOI: 10.20510/ukjpb/5/i2/155972

[25] Manavalan P, Minja L, Wanda L, et al. “It’s because I think too much”: Perspectives and experiences of adults with hypertension engaged in HIV care in northern Tanzania. PloS One. 2020; 15(12): e0243059. DOI: 10.1371/journal.pone.0243059 33270765 PMC7714125

[26] Whelton PK, Carey RM, Aronow WS, et al. 2017 ACC/AHA/AAPA/ABC/ACPM/AGS/APhA/ASH/ASPC/NMA/PCNA guideline for the prevention, detection, evaluation, and management of high blood pressure in adults: Executive summary: A report of the American College of Cardiology/American Heart Association task force on clinical practice guidelines. Hypertens Dallas Tex 1979. 2018; 71(6): 1269–1324. DOI: 10.1161/HYP.0000000000000066 29133354

[27] Bovet P, Gervasoni JP, Mkamba M, Balampama M, Lengeler C, Paccaud F. Low utilization of health care services following screening for hypertension in Dar es Salaam (Tanzania): A prospective population-based study. BMC Public Health. 2008; 8(1): 407. DOI: 10.1186/1471-2458-8-407 19087300 PMC2615777

[28] Kotwani P, Balzer L, Kwarisiima D, et al. Evaluating linkage to care for hypertension after community-based screening in rural Uganda. Trop Med Int Health TM IH. 2014; 19(4): 459–468. DOI: 10.1111/tmi.12273 24495307 PMC4118739

[29] Naanyu V, Vedanthan R, Kamano JH, et al. Barriers influencing linkage to hypertension care in Kenya: Qualitative analysis from the LARK Hypertension Study. J Gen Intern Med. 2016; 31(3): 304–314. DOI: 10.1007/s11606-015-3566-1 26728782 PMC4762819

[30] Magobe NBD, Poggenpoel M, Myburgh C. Experiences of patients with hypertension at primary health care in facilitating own lifestyle change of regular physical exercise. Curationis. 2017; 40(1): 1679. DOI: 10.4102/curationis.v40i1.1679 28470072 PMC6091569

[31] Abdeslam EK, Ahmed C, Kamal K, et al. Physical activity level and sedentary time determinants among Moroccan hypertensive patients [published online ahead of print, 2023 May 31]. Ann Cardiol Angeiol (Paris). 2023; 72(4): 101607. DOI: 10.1016/j.ancard.2023.101607 37269806

[32] Najjuma JN, Brennaman L, Nabirye RC, et al. Adherence to antihypertensive medication: An interview analysis of southwest Ugandan patients’ perspectives. Ann Glob Health. 2020; 86(1): 58. DOI: 10.5334/aogh.2904 32897274 PMC7470164

[33] Konlan KD, Afam-Adjei CJ, Afam-Adjei C, et al. Practice and sociodemographic factors influencing self-monitoring of blood pressure in Ghanaians with hypertension. Int J Chronic Dis. 2020; 2020: 6016581. DOI: 10.1155/2020/6016581 32566645 PMC7301236

[34] Herbst AG, Olds P, Nuwagaba G, Okello S, Haberer J. Patient experiences and perspectives on hypertension at a major referral hospital in rural southwestern Uganda: A qualitative analysis. BMJ Open. 2021; 11(1): e040650. DOI: 10.1136/bmjopen-2020-040650 PMC7789452 33408202

[35] Iwelunmor J, Airhihenbuwa CO, Cooper R, et al. Prevalence, determinants and systems-thinking approaches to optimal hypertension control in West Africa. Glob Health. 2014; 10: 42. DOI: 10.1186/1744-8603-10-42 PMC4046625 24886649

[36] Wekesah FM, Kyobutungi C, Grobbee DE, Klipstein-Grobusch K. Understanding of and perceptions towards cardiovascular diseases and their risk factors: A qualitative study among residents of urban informal settings in Nairobi. BMJ Open. 2019; 9(6): e026852. DOI: 10.1136/bmjopen-2018-026852 PMC6588962 31209088

[37] Leyvraz M, Mizéhoun-Adissoda C, Houinato D, et al. Food consumption, knowledge, attitudes, and practices related to salt in urban areas in five sub-Saharan African countries. Nutrients. 2018; 10(8): 1028. Published 2018 Aug 7. DOI: 10.3390/nu10081028 30087242 PMC6116014

[38] Odusola AO, Hendriks M, Schultsz C, et al. Perceptions of inhibitors and facilitators for adhering to hypertension treatment among insured patients in rural Nigeria: A qualitative study. BMC Health Serv Res. 2014; 14: 624. DOI: 10.1186/s12913-014-0624-z 25491509 PMC4267751

[39] Ilesanmi OS, Ige OK, Adebiyi AO. The managed hypertensive: the costs of blood pressure control in a Nigerian town. Pan Afr Med J. 2012; 12: 96. 23133696 PMC3489397

[40] Atinga RA, Yarney L, Gavu NM. Factors influencing long-term medication non-adherence among diabetes and hypertensive patients in Ghana: A qualitative investigation. PloS One. 2018; 13(3): e0193995. DOI: 10.1371/journal.pone.0193995 29590156 PMC5874015

[41] Nashilongo MM, Singu B, Kalemeera F, et al. Assessing adherence to antihypertensive therapy in primary health care in Namibia: Findings and implications. Cardiovasc Drugs Ther. 2017; 31(5): 565–578. DOI: 10.1007/s10557-017-6756-8 29032396 PMC5730630

[42] Osamor PE. Social support and management of hypertension in south-west Nigeria. Cardiovasc J Afr. 2015; 26(1): 29–33. DOI: 10.5830/CVJA-2014-066 25784314 PMC4392208

[43] Muthuri SK, Oti SO, Lilford RJ, Oyebode O. Salt reduction interventions in sub-Saharan Africa: A systematic review. PLoS ONE. 2016; 11(3): e0149680. DOI: 10.1371/journal.pone.0149680 26963805 PMC4786148

