# Supplementary material for: Barriers and Facilitators to Risk Reduction of Cardiovascular Disease in Hypertensive Patients in Nigeria
Source: Ann Glob Health. 2023 Dec 13;89(1):88. doi: 10.5334/aogh.4131 (PMC10723016; doi:10.5334/aogh.4131)
Supplement: Supplement. — Table S1 Summary of survey domains, survey themes, and examples of participant responses. [file agh-89-1-4131-s1.pdf]

SUPPLEMENT

Table S1: Summary of survey domains, survey themes, and examples of participant responses

STUDY DOMAINS	THEMES	PARTICIPANT RESPONSES
**PARTICIPANT BELIEFS, PERCEPTIONS, & KNOWLEDGE ABOUT HYPERTENSION** ***(SURVEY ITEMS 1-14)***	“Worrying and overthinking” as a cause for uncontrolled hypertension.	*“Lack of relaxing my mind/ overthinking. (Female, 59yo)”*<br><br>*“It is a silent killer. It comes from thinking too much and worrying too much about different issues. It has no visible symptoms. (Female, 72yo)”*
	Medication adherence and lifestyle modification are important for hypertension control.	*“Taking my medications dutifully. I don't play with it. (Female, 55yo)”*<br><br>*“I use my drugs diligently and faithfully (Female, 55yo)”*<br><br>*“My BP is high. It is a condition that needs dietary management and medication. (Female, 63yo)”*<br><br>*“… someone who has hypertension has to take it easy, reduce stress and anxiety (Female, 76yo)”*
**FACILITATORS** ***(SURVEY ITEMS 38-44)***	Family support and encouragement.	*“Once the family is aware of the health status, they help to prepare it adequately. (Male, 54yo)”*<br><br>*“Because my wife cooks my food and she knows about my condition, she makes a conscious effort to reduce the salt (Male, 43yo)”*<br><br>*“I have grandchildren and children that I can send on errands to quickly get the drug (Female, 81yo)”*<br><br>*“I don’t want to have complications and burden my family. (Male, 54yo”*
	Importance of creating a routine and incorporating lifestyle modifications into daily activities.	“I *normally do a lot of trekking…(Female, 72yo)*”<br><br>“I trek a lot. I*t helps to keep me*

		in shape and also maintain a healthy posture and a good weight (Male, 71yo)"<br><br>*"Daily usage as soon as I wake up because when I think of my health, it becomes a regimen. Take it faithfully as recommended by the doctor. (Male, 75yo)"*
**BARRIERS**<br>***(SURVEY ITEMS 45-49)***	Pain and discomfort with exercise	*"Serious body pain, due to the stress of trekking. (Female, 40yo)"*<br><br>"*I can't stand for 2-3 hours in a day as recommended by the doctor. The exercise is too strenuous for me….The walking is not easy*. *(Female, 51yo)"*
	Tasteless Meals	*"While making the family meal, I need to consider family members eating the food and for me to cook a separate meal for myself can be a bit difficult (Female, 58yo)"*<br><br>*"I prepare my food with very little salt, and everything is always okay. But when I eat other people's food with plenty salt, my legs start to swell (Female, 53yo)"*
	High cost and difficulty acquiring and taking necessary medications.	*"Unavailability of finance to buy the drugs and take it. (Female, 48yo)"*<br><br>*"It is difficult to find and expensive. Some people also sell fake ones (Female, 77yo)"*<br><br>*"The medication is plenty, taking about 10 tablets at once is not convenient (Female, 61yo)"*<br><br>*"I use it most times, but I stop when I feel better (Female, 54yo)"*
